# Use of Additive Technologies in Surgical Treatment of Chronic Posterior Dislocations of the Shoulder

**DOI:** 10.17691/stm2022.14.3.06

**Published:** 2022-05-28

**Authors:** D.V. Pavlov, S.B. Korolev, A.Yu. Kopylov, A.A. Zykin, R.O. Gorbatov, T.V. Illarionova, V.V. Gorin, R.V. Alyev

**Affiliations:** Traumatologist-Orthopedist, Traumatological and Orthopedic Department, Institute of Traumatology and Orthopedics, University Clinic; Privolzhsky Research Medical University, 10/1 Minin and Pozharsky Square, Nizhny Novgorod, 603005, Russia; Professor, Kolokoltsev Department of Traumatology, Orthopedics and Neurosurgery; Privolzhsky Research Medical University, 10/1 Minin and Pozharsky Square, Nizhny Novgorod, 603005, Russia; Traumatologist-Orthopedist, Traumatological and Orthopedic Department, Institute of Traumatology and Orthopedics, University Clinic; Privolzhsky Research Medical University, 10/1 Minin and Pozharsky Square, Nizhny Novgorod, 603005, Russia; Head of the Traumatological and Orthopedic Department, Institute of Traumatology and Orthopedics, University Clinic; Privolzhsky Research Medical University, 10/1 Minin and Pozharsky Square, Nizhny Novgorod, 603005, Russia; Associate Professor, Kolokoltsev Department of Traumatology, Orthopedics and Neurosurgery; Head of the Laboratory of Additive Technologies, Institute of Traumatology and Orthopedics, University Clinic; Privolzhsky Research Medical University, 10/1 Minin and Pozharsky Square, Nizhny Novgorod, 603005, Russia; Junior Researcher, Institute of Traumatology and Orthopedics, University Clinic; Privolzhsky Research Medical University, 10/1 Minin and Pozharsky Square, Nizhny Novgorod, 603005, Russia; Traumatologist-Orthopedist, Traumatological and Orthopedic Department, Institute of Traumatology and Orthopedics, University Clinic; Privolzhsky Research Medical University, 10/1 Minin and Pozharsky Square, Nizhny Novgorod, 603005, Russia; Traumatologist-Orthopedist, Traumatological and Orthopedic Department, Institute of Traumatology and Orthopedics, University Clinic; Privolzhsky Research Medical University, 10/1 Minin and Pozharsky Square, Nizhny Novgorod, 603005, Russia

**Keywords:** posterior dislocation of the humerus head, osteochondral injury of the humerus head, reverse Hill–Sachs lesion, shoulder joint replacement arthroplasty, 3D printing, additive technologies, McLaughlin procedure

## Abstract

**The aim of the study** was to evaluate the efficiency of additive technologies in surgical treatment of patients with osteochondral defects of the humeral head articular surface against the background of chronic posterior dislocation of the shoulder by means of comparing clinical and radiological results with the McLaughlin procedure.

**Materials and Methods:**

A prospective randomized comparative group clinical study was conducted, which included 20 patients who in 2019–2021 underwent surgical treatment of chronic posterior dislocation of the shoulder in the Traumatological and Orthopedic Department of the Institute of Traumatology and Orthopedics of the Privolzhsky Research Medical University (Nizhny Novgorod, Russia). Depending on the type of surgery, all patients were divided into 2 groups: group 1 (n=10) was subject to McLaughlin procedure, whereas group 2 (n=10) — to reconstruction of the humeral head using a customized implant based on additive technologies (3D printing). To assess postoperative results, 6 months after the surgery all patients underwent the following procedures: X-ray imaging of the shoulder joint in two projections, CT scanning, and angulometry as well as provided their responses in line with the following questionnaires: Visual Analog Scale (VAS), Disabilities of the Arm, Shoulder and Hand (DASH), American Shoulder and Elbow Surgeons Shoulder Score (ASES), Constant Shoulder Score (CSS), Shoulder Rating Questionnaire (SRQ), and the Hospital for Special Surgery Shoulder Surgery Expectations Survey (Survey of patient, SP).

**Results:**

Both the McLaughlin procedure and the reconstruction of the humeral head using a customized implant made using additive 3D printing technologies increased the range of motion in the shoulder joint, mitigated the pain syndrome and improved the patients’ quality of life. During the postoperative period, there were no infectious complications in both groups. The total bed-day in group 1 was 7 [5; 9] days; in group 2, it was 8 [6; 9] days. There was no recurrence of dislocation or progression of osteoarthritis of the shoulder joint in patients in both groups during 6 months after the surgery. The ASES, SP, SRQ, CSS, DASH, and VAS questionnaires assessment for both groups showed a statistically significant improvement for all indicators in the postoperative period. There were no statistically significant differences found between the groups as to the results of angulometry and answering the questionnaires.

**Conclusion:**

Customized implants made using additive technologies can shorten the surgery duration by 1.3 times, whereas the volume of intraoperative blood loss — by at least 1.5 times compared to the McLaughlin procedure.

## Introduction

Shoulder dislocations are among the most common musculoskeletal injuries with an incidence of 17 cases per 100,000 persons per year [1]. Chronic posterior dislocations amount to 23% of all shoulder dislocations [2]. This pathology is characterized by chronic pain syndrome and severe dysfunction of the upper extremity. Shoulder dislocation is chronic if it is not cured within 3 weeks [3]. 60–70% posterior dislocations of the shoulder occur in men aged 20 to 50 years [4]. In 65% of cases, such dislocations are followed by bone defects in the proximal humerus [5]. Moreover, osteochondral defects of this localization are diagnosed in 86% of patients with recurrent shoulder instability (reverse Hill–Sachs lesion) [6].

One of the most important factors to influence the choice of surgical treatment tactics is the size of the humeral head osteochondral defect in reverse Hill– Sachs lesion. For smaller defects (up to 25% of the joint surface), it is recommended to conduct a modified McLaughlin procedure being transposition of the lesser tubercle followed by attachment of the subscapularis muscle tendon and its fixing in the impressed point of the humerus head. In case of moderate and severe defects of the bone tissue (over 25% of the joint surface), it is recommended to perform bone allografting or arthroplasty of the shoulder joint [7].

Yearly, there is an increase in the number of patients treated using additive technologies. 3D printing provides for making customized high-precision implants that can replace bone defects of any shape, complexity and size. The use of additive technologies reduces the volume of blood loss, shortens the surgery, and improves the results of patient treatment [8, 9].

The choice of the most appropriate treatment option for this pathology is complex and involves multiple factors. Despite the issue relevance, the available literature sources provide no results of comparative studies of surgical treatment of patients with humerus head defects against the background of chronic dislocation of the shoulder using the McLaughlin procedure and customized implants made on a 3D printer.

**The aim of the study** was to evaluate the efficiency of additive technologies in surgical treatment of patients with osteochondral defects of the articular surface of the humeral head against the background of chronic posterior dislocation of the shoulder by means of comparing clinical and radiological results with the McLaughlin procedure.

## Materials and Methods

There was a prospective randomized clinical trial conducted; it enrolled 20 patients (17 men and 3 women) who underwent surgical treatment for chronic posterior shoulder dislocation during the period of 2019–2021 in the Traumatological and Orthopedic Department of the Institute of Traumatology and Orthopedics of the Privolzhsky Research Medical University (Nizhny Novgorod, Russia). Surgery on the right shoulder joint were performed for 14 patients, on the left — for 6 patients.

The major complaints in all patients included pain syndrome, a significant movement limitation in the shoulder joint, and impaired self-care functions.

Inclusion criteria: a history of the chronic posterior dislocation of the shoulder, a defect in the humerus head ranging in size from 10 to 45% of the bone, the patient’s consent to participate in the study. Exclusion criteria: age less than 18 and over 80 years, stage III of the shoulder joint arthrosis, deformation of the glenoid cavity of the blade bone, signs of severe concomitant diseases (hematological, immunological, urogenital, endocrine, psychiatric, cardiovascular, dermatovenereological, neurological, and other diseases).

**Figure 1.**
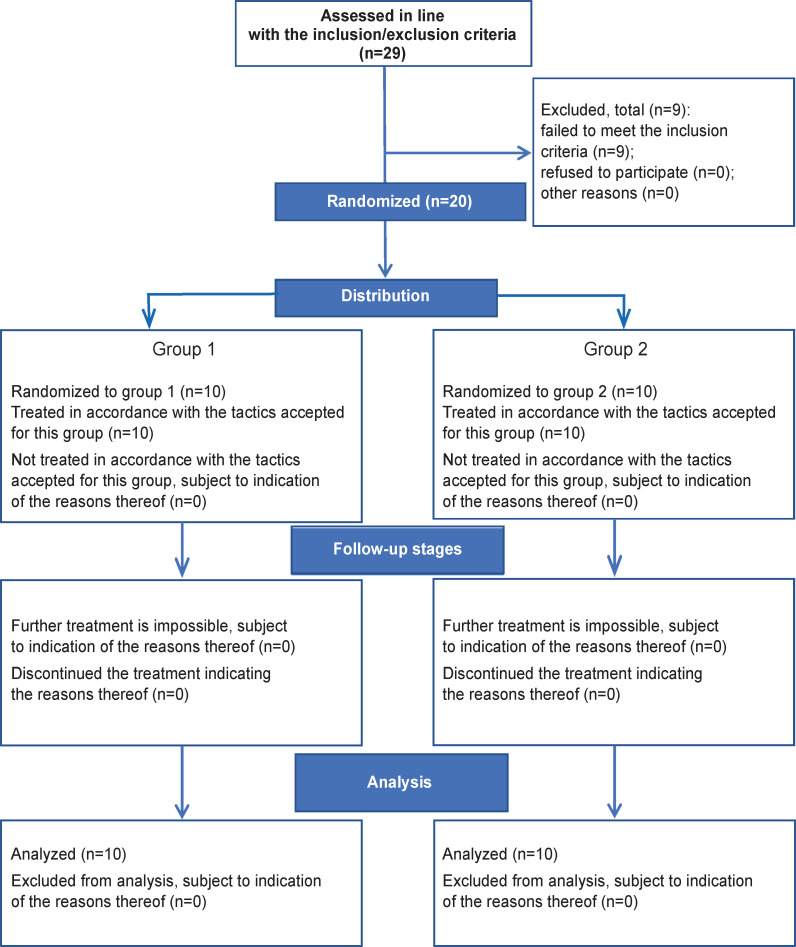
Study design

The study participants were divided into two groups depending on the type of surgical intervention: patients from group 1 (n=10) underwent the McLaughlin procedure, whereas patients from group 2 (n=10) had reconstruction of the humeral head with a customized implant made using additive technologies (3D printing). Randomization was conducted by means of sequentially numbered envelopes. The study design was developed in accordance with the CONSORT international recommendations [10] (Figure 1).

Patients’ characteristics for both groups are shown in Table 1. There were no statistically significant (p>0.05) differences between them in terms of age, time passed after injury and surgery, and the VAS indicators.

**Table 1:** Patients’ characteristics, Me [25; 75]

Indicator	Group 1	Group 2
Age (years)	51.5 [38; 65]	52.4 [33; 61]
Time after injury (months)	4.1 [2; 9]	5.0 [3; 11]
Time after surgery (months)	6.0 [4; 8]	6.0 [3; 9]
VAS before surgery	8.0 [5; 10]	8.0 [4; 11]

At the stage of pre-surgery preparation and 6 months after the surgery, all patients underwent angulometry and filled in the following questionnaires: Visual Analog Scale (VAS), Disabilities of the Arm, Shoulder and Hand (DASH) [11], American Shoulder and Elbow Surgeons Shoulder Score (ASES) [12], Constant Shoulder Score (CSS) [13], Shoulder Rating Questionnaire (SRQ) [14], and the Hospital for Special Surgery Shoulder Surgery Expectations Survey (Survey of Patient, SP) [15].

The size of the humeral head defect in patients of group 1 and group 2 was 28±2 and 36±4% (p<0.05) respectively, which corresponds to moderate defects.

Before surgery, all patients experienced movement limitations in the shoulder joint (up to 10±5°) and had a pronounced pain syndrome, their injured upper extremity was immobilized with a scarf bandage, but they had no vascular and neurological abnormalities. The range of motion in the elbow, wrist and hand joints was within the normal range in all patients.

At the stage of pre-surgery preparation and 6 months after the surgery, all patients had ultrasound dopplerography of the vessels of the upper limbs, radiography in two projections, and CT scanning of the shoulder joint.

A customized implant was made on a 3D printer from the titanium alloy in line with the data from the CT scan of the shoulder joint.

***The McLaughlin procedure*** was performed using the combined anesthesia: blocking of the brachial plexus and inhalation endotracheal anesthesia. The patient was placed on the surgical table in a semi-sitting position (“beach chair”). The osteotomy of the lesser tubercle was performed intermuscularly and deltopectorally without cutting off the tendon of the subscapularis muscle from it. Then the humerus dislocation was cured which revealed its head and the reverse Hill–Sachs lesion. Decortication and osteoperforation of the impression area were followed by replacement of the humerus head defect with an autograft of the lesser tubercle and its further osteosynthesis with two cannulated screws. After that, transarticular fixation of the shoulder joint was performed using the Kirschner wires.

**Figure 2.**
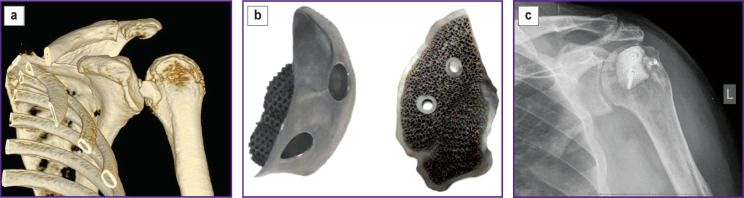
Replacement of the humerus head defect with a customized implant: (a) a CT scan of the right shoulder joint before surgery; (b) a 3D-printed customized implant; (c) an X-ray image of the left shoulder joint after surgery

***The surgery with a customized implant made using 3D printing*** was performed using combined anesthesia: blocking of the brachial plexus and inhalation endotracheal anesthesia. The patient was placed on the surgical table in a semi-sitting position (“beach chair”). The osteotomy of the lesser tubercle was performed intermuscularly and deltopectorally with cutting off the tendon of the subscapularis muscle from it. Then dislocation of the humerus head was cured with release and mobilization of the shoulder joint. Decortication and osteoperforation of the impression area were followed by replacement of the humerus head defect with a customized implant and its fixation with 2–3 screws (Figure 2). Then the subscapularis muscle was refixed and the shoulder joint was transarticularly fixed using the Kirschner wires.

During the post-surgery period, all patients got their surgically operated upper extremity immobilized with a Dezo soft bandage for 5–6 weeks. The wires were removed 4 weeks after the procedure.

The study was conducted in accordance with the Helsinki Declaration (2013) and approved by the Ethics Committee of the Privolzhsky Research Medical University. Each patient provided the informed consent to participate in the study.

**Statistical analysis** of the results was performed using the Statistica 10.0 software. The pattern of the characteristics distribution was assessed using Shapiro– Wilk normality test. Taking into account asymmetric distribution, the data were presented as a median, 25^th^ and 75^th^ percentiles. The groups were compared using the Mann–Whitney test. Differences were considered statistically significant at p<0.05 (subject to Bonferroni adjustment for multiple comparisons, p≤0.017).

## Results and Discussion

At the control examination 6 months after the surgery, CT scanning and X-ray imaging of the shoulder joint revealed no signs of aseptic necrosis or instability of the customized implant in all patients of group 1 and group 2.

The patients had no infectious complications during the post-surgery period. The wounds got healed by primary intention, the sutures were removed on day 14–16. The total amount of bed-days in group 1 was 7 [5; 9] days; in group 2, it was 8 [6; 9] days. There were no statistically significant differences for this indicator (p>0.05).

The length of the surgical operation in patients of group 1 was 100 [80; 120] min, of group 2 — 1.3 times shorter (75 [60; 90] min) (p<0.05). The decrease in surgical intervention time in patients using additive technologies in comparison with the McLaughlin procedure is due to the precise correspondence of the customized implant to the parameters of the bone defect, as well as to its faster positioning.

The volume of blood loss in patients of group 1 was twice as high as in group 2 and amounted to 200 [100; 250] ml, whereas in group 2 it was 100 [80; 120] ml, respectively (p<0.05). The decrease in blood loss in patients who were treated using a customized implant is believed to be related to lack of need for osteotomy of the lesser tubercle of humeri and a shorter surgical intervention.

Patients from both groups showed no recurrence of dislocation or progression of osteoarthritis of the shoulder joint during 6 months post-surgically.

Angulometry indicators improved statistically significantly (p<0.05) during the post-surgical period in all patients. At that, comparison of the range of motion in the shoulder joint showed no statistically significant differences between the two groups of patients (p>0.05) (Table 2).

**Table 2:** Angulometry results (degrees), Ме [25; 75]

Movement	Group 1	Group 2
Bending	151 [150; 153]	145 [140; 150]
Abduction	151 [146; 153]	140 [120; 150]
External rotation	25 [21; 35]	30 [25; 35]
Internal rotation	80 [55; 90]	85 [77; 90]

According to the results of the patients’ questioning with the ASES, SP, SRQ, CSS, DASH, and VAS questionnaires, there was a statistically significant (p<0.01) improvement in all indicators observed during the post-surgical period. At that, comparison of the data showed no statistically significant differences between the two groups of patients (p>0.05) (Table 3).

**Table 3:** Results of the patients’ questioning before and after surgery (points), Ме [25; 75]

**Questionnaire**	Group 1		Group 2	
	Before surgery	After surgery	Before surgery	After surgery
	1	2	3	4
ASES	23 [18; 26]	92 [84; 95]	28 [19; 35]	85 [48; 92]
SP	83 [82; 85]	70 [69; 73]	83 [83; 85]	68 [66; 75]
SRQ	31 [23; 39]	63 [62; 65]	25 [22; 30]	52 [40; 61]
CSS	28 [18; 33]	80 [67; 82]	23 [21; 29]	62 [48; 71]
DASH	73 [65; 77]	2 [2; 26]	71 [66; 80]	5 [3; 30]
VAS	8 [8; 9]	1 [0; 3]	8 [7; 9]	0 [0; 0]

Note: p_1–2_<0.017; p_3–4_<0.017; p_2–4_>0.017.

The analysis of the current national and foreign literature sources failed to find studies on assessment of long-term results of patients treatment after replacement of humerus head defects against the background of chronic posterior dislocation of the shoulder with customized implants made using additive technologies. There is information on reconstruction of the humerus and the glenoid blade surface defects using additive technologies and on complete replacement of the humerus in cancer patients. For example, Hu et al. [16] demonstrate the results of using customized implants in combination with a 3D-printed glenoid element of endoprosthesis in patients after the humerus resection due to a tumor. After surgery, most patients showed a significant mitigation in pain syndrome and improved quality of life.

The McLaughlin procedure is generally used for small to moderate defects in the humerus head. It proved to be an intervention with a low risk of complications and a good functional result [7, 17]. However, most patients have limited external rotation in the post-surgery period, which was confirmed both in this study and in articles of other Russian authors. For example, Belyak et al. [7] demonstrated data on surgical treatment of 7 patients with posterior chronic impacted incomplete dislocations of the shoulder joint. Post-surgery results in all patients were rated as good and satisfactory. External rotation in the surgically operated shoulder joint was 24±6° [7], whereas in this study it reached 25±8°.

Defects in the humerus head were greater in the group of patients with implants made using additive technologies than in patients of group 1. Regardless of this fact, the indicators according to the questioning results in the post-surgery period did not differ significantly, which confirms clinical efficiency of additive technologies even with large bone defects. However, to specify the advantages of this surgical treatment method compared to the traditional ones additional comparative studies on larger samples of patients are definitely required.

Thus, development of additive technologies provided for making customized precision implants to replace bone defects of the humerus head. They allow complete restoration of the structure and functioning of the shoulder joint and highly correspond to the volumetrics of the damaged area.

## Conclusion

Reconstruction of the humerus head using customized implants made with additive technologies increases the range of motion in the shoulder joint, reduces pain syndrome and improves the patients’ quality of life. The use of customized 3D-printed implants provides for shortening the surgical intervention by 1.3 times and reduction of intraoperative blood loss by 2 times compared to the McLaughlin procedure.
